# Near-peer education: a novel teaching program

**DOI:** 10.5116/ijme.5738.3c28

**Published:** 2016-05-30

**Authors:** Sara de Menezes, Daphne Premnath

**Affiliations:** 1 Faculty of Medicine Nursing and Health Sciences, Monash University, Australia

**Keywords:** Near-peer education, peer-to-peer teaching, medical education, Objective Structured Clinical Examination (OSCE), Australia

## Abstract

**Objectives:**

This study aims to: 1) Evaluate whether a near-peer program improves perceived OSCE performance; 2) Identify factors motivating students to teach; 3) Evaluate role of near-peer teaching in medical education.

**Methods:**

A near-peer OSCE teaching program was implemented at Monash University’s Peninsula Clinical School over the 2013 academic year. Forty 3rd-year and thirty final-year medical students were recruited as near-peer learners and educators, respectively. A post-program questionnaire was completed by learners prior to summative OSCEs (n=31), followed by post-OSCE focus groups (n=10). Near-peer teachers were interviewed at the program’s conclusion (n=10). Qualitative data was analysed for emerging themes to assess the perceived value of the program.

**Results:**

Learners felt peer-led teaching was more relevant to assessment, at an appropriate level of difficulty and delivered in a less threatening environment than other methods of teaching. They valued consistent practice and felt confident approaching their summative OSCEs. Educators enjoyed the opportunity to develop their teaching skills, citing mutual benefit and gratitude to past peer-educators as strong motivators to teach others.

**Conclusions:**

Near-peer education, valued by near-peer learners and teachers alike, was a useful method to improve preparation and perceived performance in summative examinations. In particular, a novel year-long, student-run initiative was regarded as a valuable and feasible adjunct to faculty teaching.

## Introduction

Peer-education is a widely-used teaching model where students teach their peers and junior students. A near-peer teacher (NPT) is someone at least one year senior to their near-peer learner (NPL) in the context of medical training.^1^^,^^2^

Peer-to-peer teaching was first most clearly proposed in 1988 by Whitman and Fife,^3^ who explored peer-education strategies used in tertiary education. As early as the 1950s, undergraduate students had been employed as teaching and laboratory assistants to save professorial time and limit lecture-based didactic learning.^1^^-^^5^

Gaining popularity in recent years, the mutual benefit of peer-education is supported by subjective and objective evidence. Learners find that peer-education improves their understanding of subject matter and is targeted at an appropriate level. Other benefits include better access to role-models, a ‘safer’ learning environment, improved confidence and enhanced motivation. Furthermore, some studies show that peer-educators are occasionally preferred over faculty educators.^6^^-^^9^

The perceived benefits for peer-educators are also identified, albeit in less detail. Some suggest teachers’ motivation to learn and understand subject matter are enhanced through the process of teaching.^5^^,^^6^ In addition to improving their own learning, teaching develops communication, leadership, organizational and group facilitation skills.^5^^,^^7^ Furthermore, peer-education supplements professional and personal development.^5^^,^^7^ The skills developed as a peer-teacher were also found to assist medical students fulfil their roles as educators when they became junior doctors and later physicians.^2^^,^^5^^,^^6^

The Objective Structured Clinical Examinations (OSCEs) are a widely-used assessment tool in medical education. They are used to examine the practice and integration of history-taking, physical examination and interpretation of medical investigations. Existing near-peer teaching programs (NPTPs) are targeted at teaching clinical skills in isolation rather than strategies required for OSCEs and the art of integration of clinical skills.^7^^,^^10^^-^^12^ Only one study by Rashid et.al.^1^ attempted to prepare students for summative examinations. In addition, while short-term, peer-led programs have been described, literature review did not reveal programs which provided consistent teaching over a sustained period of time.^1^^,^^13^^-^^19^ Finally, while there have been suggestions made for why peer-teachers volunteer for their role,^3^ few studies explore peer-teachers’ motivation in depth.

In order to bridge this gap, this paper describes a pilot near-peer program conducted over 10 months at Peninsula Clinical School (PCS) in 2013 which aimed to improve exam performance of Year 3 medical students.

The authors are alumni of Monash University, and were final year medical students at the time this teaching program was conducted at PCS. Anecdotal evidence prior to commencing the program suggested junior students yet to complete OSCEs and senior students who had recently completed OSCEs did not feel adequately prepared for their summative examinations. In 2013, as there was also limited near-peer teaching provided at MUPCS.

Therefore, the authors hope this paper will provide valuable insight into existing knowledge of peer-led educational programs and help establish the efficacy of long-term peer-education in improving exam performance. This project was conducted with the aim of answering the following key questions:

        1. Does a structured peer-led education program improve perceived OSCE performance?

        2. What motivates people to teach others?

        3. Is peer-led teaching a valuable addition to the medical school curriculum?

## Methods

### Study design and participants

A NPTP was implemented at Monash University’s Peninsula Clinical School (MUPCS) at Frankston Hospital, Victoria in Australia, to assist Year 3 medical students with preparation for their summative OSCEs. The program was devised by final year students, based on the university curriculum, faculty resources and their own experiences of OSCE preparation. Ethics approval was granted for this project by the Monash University Human Research Ethics Committee (MUHREC) in 2013.

The PCS OSCE Teaching Program (POTP) was conducted on a cohort of MUPCS medical students in 2013 over one academic year, with faculty support. Near-peer learners (NPLs) consisted of 40 third-year medical students based at MUPCS, who were recruited via email and social media.

Final-year medical students rotating through MUPCS in 6-week placements were similarly invited via email and social media to participate as near-peer teachers (NPTs). The 30 NPTs recruited were allocated into groups of 4 - 6 and scheduled to teach for a minimum of 6 weeks, depending on the number of rotations they chose to participate in.

A curriculum guide was provided to NPTs to ensure continuity and diversity in topics as NPTs changed every 6 weeks. This allowed NPLs to achieve a sense of progression on a variety of topics throughout the year. A bank of OSCE stations including previous examples of faculty OSCEs, published OSCEs from textbook and online resources, as well as OSCEs created by the authors reflecting the style of summative OSCEs was made available to NPTs. The NPTs were then encouraged to create their own OSCEs, based on their selected topic for the week and own recent experiences of summative OSCtEs, which were added to this bank. NPTs were also required to create a worksheet for NPLs for each session. These contained key learning points for the session and served as a revision tool.

Weekly sessions were led by the group of NPTs over 1.5 hours. Each session consisted of two mock OSCE stations for participants to complete in groups of three as ‘patient’, ‘student’ and ‘examiner’ under strict time limits reflective of third-year summative exams. A mock OSCE stem was provided to the NPL while a patient history was provided to the ‘patient’ and the ‘examiner’ a mock examination sheet. This was timed according to faculty guidelines (2 minutes of preparation, 8 minutes being examined and answering examiner questions.  At the end of the 8 minutes, NPTs provided feedback individually and to the group where possible. This was followed by a short teaching session by NPTs based around the worksheets they created. The POTP ran from March 2013 until November 2013, ending just prior to the NPLs’ final OSCE exams.

### Data collection

Qualitative data was collected from both NPTs and NPLs. The NPLs initially completed an 11-item questionnaire prior to the NPLs sitting for their final exam. Open-ended questions were used to assess NPLs’ opinions regarding strengths and weaknesses of the teaching program as well as the perceived value of the near-peer intervention in their exam preparation. After completion of their summative OSCEs, NPLs were then invited via emails and social media to participate in focus groups, to assess if their opinions of the teaching program differed post-exam and their perspectives on the value of near-peer education. NPTs were similarly invited to individual interviews, in which questions were directed to their experiences teaching in the program as well as perspectives on near-peer education.   The different modalities of data collection were necessary to accommodate participant and researcher time and resource constraints, which was conducted in university holidays.

### Data analysis

Qualitative data was collected and analysed with the view of answering the research questions proposed in the aims of this paper. In order to assess the perceived benefit towards summative assessment, areas assessed include program content, structure, teaching style, logistics and perceived utility. These results were compared in data collected before and after the summative OSCEs to see if opinions had changed. NPT interview responses were then further evaluated for consistent and emerging reasons for motivations for students to teach and perspectives of the near-peer teaching program.

## Results

About 31 NPLs completed the pre-exam questionnaire. This represented 78% of the NPLs who attended the NPTP sessions throughout the year. In this cohort, there was an over-representation of undergraduate students (90%) and the majority of these were in the 18 to 21 year age group (n=18).  Furthermore, there were no students who did not attend at least one teaching session, while 48% of students attended more than 10 sessions.

There were then 22 NPLs who completed questionnaires agreed to be contacted for post-OSCE follow-up. One third of these students participated in the post-exams focus groups (n=7), of whom most attended at least 10 teaching sessions.

Of the 30 NPTs who were invited to participate, one third agreed to attend individual post-program interviews. The demographics of the NPTs were comparable to that of the NPLs, where majority (70%) of NPTs were undergraduate students. It was noted that the number of sessions NPTs attended correlated with time spent rotating through MUPCS, indicating that without exception, NPTs chose to participate in the POTP each time they were rotated to Frankston Hospital. Furthermore, all NPTs interviewed reported having previous experience in teaching. 

Overall, peer-learners unanimously felt the POTP was realistic and had utility. They believed it was true to the format of actual OSCEs which had a positive impact on their exam preparation and performance. This was reflected by students both before and after completion of summative examinations.

Evaluation by peer-learners of program content consistently highlighted it was relevant to exams and at an appropriate level of difficulty. Senior peers appeared to “know what level the information needs to be at” and deliver it such that was applicable to third-year course requirements. Learners perceived their senior peers to have “firsthand experience” of OSCEs which was both current and aimed at successful exam performance.  Approximately 70% of peer-learners felt content covered in POTP was an accurate representation of the summative OSCEs. In comparison, learners expressed faculty teaching by senior clinicians could be, at times, less exam-oriented and discussed content uncommonly examined in practical assessments. They additionally felt faculty lecturers, tutors and hospital-based clinicians had different interpretations of curriculum content and requirements, which did not assist with exam preparation. This view was unchanged after the formal OSCE examinations, where NPLs in focus groups unanimously agreed content was appropriately chosen and taught. A minority of NPLs, however, identified topics which they felt were not covered in the POTP such as “Occupational Health” and “Pathology”. However, these were also the topics most learners felt were generally not covered well in the formal curriculum.

Peer-learners also valued the structure of the program, particularly the reinforcement of clinical skills provided by exam-style mock OSCEs consistently over 10 months. Weekly practice in an environment recreating exam conditions trained students to work within a time limit. They unanimously felt this assisted them in being systematic and efficient in summative OSCE stations. In addition, they felt that this buffered them against the time pressure faced in the actual summative assessments. Students further identified regular reinforcement of learnt knowledge and skills with senior guidance helped to improve their confidence approaching their final exams. Learners expressed feeling less stressed prior to their exams, as they felt participation in the program helped them structure their learning throughout the year. This subsequently led to feelings of preparedness prior to summative assessments.

Themes pertaining to mentorship between peer-learners and peer-teachers were also identified to add value to the teaching program. The NPLs reported observing less hierarchical barriers between themselves and NPTs, even outside of the learning environment. They admitted to being more comfortable asking questions in sessions since they felt less intimidated being taught by their peers. Moreover, NPTs were regarded as more empathetic since they could relate to the challenges of being in the NPLs positions themselves. The peer-teachers admitted an inclination to providing mentorship to their learners when they took on educational roles. This was interestingly reflected by the learners themselves who preferentially sought out their NPTs outside the teaching sessions.

Analysis of peer-teacher interviews identified several key reasons as motivators to teach. As a whole, peer-educators who participated in this POTP unanimously felt teaching others produced mutual benefits and a sense of fulfilment. Peer-teachers identified it required greater familiarity and understanding of a topic in order to teach it well. Therefore, their involvement in the program was considered a form of revision and method to consolidate their own knowledge. They felt this was especially important as they transitioned into their role as junior doctors. The NPTs felt questions asked by learners challenged them to have deeper insight of subject matter and organise information in a way that could be taught easily. Moreover, it provided the NPTs with motivation to learn more difficult and unfamiliar topics.

Peer-educators also viewed participation in teaching activities as an opportunity to network. They viewed teaching their juniors as investing in their future colleagues. This was reinforced by the finding that both NPLs and NPTs recognized the opportunity for mentorship due to improved relationships both inside and outside the classroom. NPTs further identified teaching as contributing to their professional development. They believed taking on the role of an educator helped hone their public-speaking abilities and improve their overall confidence. In addition, simplifying and organising information to teach others was felt to improve their communication skills. Notably, peer-teachers recognised that while teaching juniors is an essential part of being a doctor, it is not a skill taught in medical school. Therefore, the NPTs involved in POTP saw this as a unique opportunity to gain essential capabilities that would contribute to their careers.

Analysis of the data also showed common themes between teachers and learners about what motivates them to volunteer to teach. Both groups identified gratitude as a strong motivator. The NPTs cited having previously benefited from teaching provided by peer-educators when they were junior, as being a driver of their involvement in POTP. Interestingly, at the end of the POTP, the exact sentiments of gratitude were expressed by NPLs in relation to the teaching they received throughout the year. About 88% of the learners admitted they would be more likely to become peer-teachers themselves as a result of this experience.

Another shared theme between NPTs and NPLs was that of gaining gratification from teaching. All the peer-teachers reported enjoying the process of teaching others. They further identified this satisfaction deriving from assisting students identify areas for improvement. This equipped them with the notion they were making a difference to their learners’ education, which further motivated their efforts. Similarly, it emerged at end of the program peer-learners who expressed interest in becoming future educators gained fulfilment from the idea of teaching others.

This POTP, which was designed by the authors, was seen as valuable to both NPLs and NPTs. They believed this was because it focused on regular teaching of clinical skills in a simulated exam environment, which was different to education delivered in lectures and bed-side tutorials. Furthermore, the program was designed to guide peer-teachers and provide them with teaching skills that would allow effective content delivery.

Overall both groups in our study agreed that near-peer education would be a useful adjunct to the medical school curriculum and in preparation for their OSCEs. Interestingly, both the NPLs and NPTs were supportive of an exam-oriented structure of teaching in their curriculum. They all felt however, that this should not replace traditional forms of teaching, which should still play a prominent role. Moreover, they felt collaboration between the faculty and peer-teaching groups may help improve the efficacy of medical school curriculums.

When the researchers explored the notion of integrating peer-led education with the medical school curriculum both groups were undivided about one thing. One hundred percent of participants agreed that the voluntary nature of the POTP was essential to its success. From the peer-learner perspective, students felt that although making the sessions mandatory would improve attendance to the sessions, it would be burdensome to have to attend yet another mandatory session on top of their other tutorials, lectures and bed-side teaching sessions. The NPLs viewed the voluntary nature of the POTP as giving them a choice to take their learning into their control by attending a voluntary education session. Moreover, word-of-mouth about the value of the program was felt by both NPLs and NPTs to boost participation as the year went on. From an NPT point of view, they regarded the did not think mandatory teaching would have the same benefits as would voluntary participation in the program. The main reason cited for this is that not everyone feels confident enough or has the skills and desire to teach others. Therefore, it was felt that there was an imposition to teach that it would affect the quality and enthusiasm of peer-educator participation.

## Discussion

The concept of “cognitive congruence” in education has been described by Lockspeiser et.al.,^4^ who observed that possessing a similar depth, complexity of knowledge and information organization is what allows NPTs to convey information effectively to NPLs.^4^ A parallel concept of “social congruence” refers to the approachability of peer-educators, who have similar capabilities as their learners compared with senior academic staff.^20^ This creates a less stressful environment where learners feel more willing to clarify presumed “basic” knowledge without fear of retribution.^5^ Older peers are also viewed as role-models who provide mentorship to facilitate socialisation into the medical environment.^5^

This was reflected in our study where peer-learners felt NPTs taught at an appropriate level of difficulty. The vast majority of NPLs also acknowledged feeling less intimidated being taught by their senior peers. These findings are thought to correlate to a low-stress environment which was conducive to optimal learning. This is consistent with many near-peer education programs and offers further support to the principle of cognitive congruence as a key reason for the effectiveness of peer-education.^4^^,^^5^^,^^8^^,^^10^^,^^20^^,^^21^

Peer-educators, being at a closer level of learning, possess more relatable organisation of knowledge which facilitates learning for their junior peers. Students also highlighted the value of being taught these strategies in organising information, building problem-solving frameworks and honing time management, which was less often addressed by faculty-led education. This could be because NPTs can recall their own experience of learning the content and share their existing memory tools. Furthermore, NPTs also have experience being examined in similar content. Therefore, they are able to guide NPLs as to what information is ‘essential knowledge’ versus ‘minutiae’ at the learners’ level.

This is supported by Whitman and Fife^3^ as well as Ten Cate and Durning^5^ who describe near-peers having “conscious competence” of a recently mastered skill, whereas expert educators possess “unconscious competence” making them less adept at assisting novices to gain understanding of a seemingly basic concept.^3^^,^^5^ Furthermore, Hall et.al.^21^ found even minimizing the degree of “cognitive distance” improved perceived usefulness and enjoyment of the teaching sessions.^21^ As a result, senior medical students rated more highly than more experienced junior doctors.^21^

### Peer-educator perspective

A universally described benefit by our program’s NPTs was that of improved understanding and deeper insight of subject matter gained from teaching. They cited revision of previously learnt medical topics as a strong motivator, especially in their transition into the medical workforce. Moreover, senior students identified the need to organise difficult concepts in a way that was simple for NPLs to understand. The NPTs recognised that this was a skill applicable both in their role as future educators as well as their interactions with patients.

It has been recognised that cognitive skills required to teach junior peers are different to those used to study the same subject for their own needs.^4^ In order to teach, educators must review subject material in adequate depth, reorganise it in a systematic manner and deliver information in a manner easily but fully grasped by learners.^4^ Examinees who have taught a subject perform better when examined on it, suggesting that “to teach is to learn twice”.^3^^,^^19^

Another unique feature of our program is that NPTs were encouraged to develop their own content based on the available bank of mock-OSCEs and their past experiences. The authors believe this encouraged the active process of distillation of core concepts that would be applied in the teaching sessions. Furthermore, this process required peer-teachers have a good understanding of the subject matter before being able to create a meaningful learning experience for their learners. The intended outcome was to teach the NPTs “how to teach” effectively, which was recognised by the participants at the end of the program.

NPTs also acknowledged positive past-experience with peer-educators as a motivator to teach others. They admitted they themselves had been taught by senior peers and gained benefit from those interactions. This left them with a sense of gratitude, duty to “pay it forward” and continue the legacy of peer-teaching, manifested by their involvement in our POTP. Interestingly, these exact sentiments were unexpectedly expressed by our NPLs at the end of the POTP. The researchers believe this reflects one of the key ways medicine propagates knowledge; through role-modelling and mentorship. Therefore, we believe peer-led programs have the potential to harness these tendencies at an early stage and create a teaching culture in doctors.

The medical profession relies on a hierarchical transmission of knowledge usually from senior doctors to junior doctors.^2^^,^^5^^,^^6^ This was supported by the NPTs in our program who recognised teaching as an important part of their role as future clinicians. However, doctors often feel unprepared due to a lack of teaching experience, difficulty communicating complex information and time management.^2^^,^^7^

To mitigate this, many NPTPs have used various supports to improve NPT confidence. This includes training, provision of resources and presence of supervising senior faculty members or clinicians to co-lead.^2^ Of note, the Students-As-Teachers (SAT) initiative in American medical universities and the Teaching-on-the-Run program in Australia are courses designed to address anxiety around taking on a teaching role.

Importantly, the NPTs involved in our program were all volunteers. It is likely therefore that those involved in POTP had an interest in teaching already or wanted to develop those skills. Remarkably, the enjoyment and satisfaction derived from teaching peers appeared directly proportional to the fact that our NPTs were acting on a voluntary basis. One hundred percent of peer-teachers admitted that if it was made mandatory to partake in teaching POTP, that it would completely change their attitude towards the task. One explanation identified is that not all senior students are interested in teaching, therefore feeling uncomfortable in this role could affect the quality of teaching. Furthermore, being “forced to teach” may limit the altruistic motivations that drove most of our volunteer NPTs to participate in the program.

### OSCE program structure and content

A review of literature demonstrates NPTPs have pre-determined content, such as “uses of ultrasound imaging” or “physical examination”.^7^ There is however some flexibility, mostly given to the choice of teaching method. A unique feature of our POTP was the freedom given to NPTs to choose weekly topics within a pre-allocated medical specialty with the broad aim of OSCE preparation. Our NPTs then chose session content based on their personal experiences of OSCEs, advice from their own mentors and fellow NPTs. Importantly, choice of topic was also guided by learners’ requests and perceived weaknesses. The NPLs subsequently felt topics chosen were relevant and exam-oriented; both before and after completing their formal OSCE examinations.

The disadvantage however of a semi-structured program is that weaknesses of NPTs may reflect gaps in knowledge of medical students across all year levels or the overall curriculum. This was reflected in responses from the Year 3 learners, who felt they would have appreciated more pathology, histology and occupational medicine mock-OSCEs, which they identified as their weaknesses. It is very likely though that these are also weaknesses of the NPTs themselves who admitted to teaching topics they “enjoyed” or were “useful”. Thus, without some measure of control over teaching content, gaps in knowledge are likely to be perpetuated if the NPTs are the sole source of practical exam preparation.

Evaluation of our results further suggests final-year students, having conducted OSCEs in the preceding two years, have more current experience than faculty educators, who may have finished their medical training many years prior. Our junior students appeared to appreciate this firsthand advice and trusted their NPTs’ experiences. This leads us to further hypothesise those students and university academics have different goals.

It appears that students prioritise successful exam performance compared to gaining knowledge applicable in the workforce, which is the reverse of faculty priorities. While our participants largely acknowledge the value of “ward-based learning” encouraged by the faculty, they agreed the knowledge and skills required to succeed in assessments was different. It may be worthwhile then considering the possibility of dissonance between the medical school curriculum and practical knowledge needed for the workforce that lend to this perception.

### Program strengths

In contrast to many other short-term peer-education programs, the POTP ran for the duration of the 2013 academic year. In fact, at the time of the writing of this paper, the POTP still exists and continues to be run by former NPLs of the program. Of note, in 2013 MUPCS provided varied learning opportunities for students, including lectures, bedside and case-based tutorials. The positive reception to the POTP and its perceived value by participants however suggests it is a useful adjunct to exam preparation. This was reinforced by the spike in NPL attendance particularly closer to summative OSCEs.

In addition to the transfer of knowledge, a strength of this program was the opportunity it gave students to regularly refine their practical skills with senior guidance. The authors believe this was an essential part of the program’s success. We believe this allowed consolidation of skills to the point where in high-stress exam environments, students gained automaticity in their approach to the exam structure. This allows students to then focus on thinking about the clinical problem rather than what the next step in the structure should be.

Another strength of the program is interestingly how the POTP today is led and run by former NPLs of the pilot program in 2013. The authors believe this is a reflection of the earlier identified themes of mutual benefit, gratification from teaching and gratitude for the experiences they had as NPLs.

Furthermore, compared to peer-education programs which exist in the literature, this teaching program devised by the authors also have some unique features including its goal to improve students’ perceived summative exam performance, duration over one year and completely student-driven initiative with minimal faculty supervision. In addition, our study has provided valuable insights into the motivations of peer-educators which was an identified gap in the literature. This could provide a basis for other medical educators to build on and consider the value of implementing similar programs.

### Program limitations

While this pilot POTP was supported by MUPCS from its inception, the researchers strived for the teaching program to be student-led to preserve the strengths of peer-led education. Notably, the teaching sessions were generally not attended by senior faculty staff and mock OSCEs were not reviewed by faculty staff. This is in contrast to most other peer-led programs, which could introduce the risk of inaccurate teaching by inexperienced teachers. This was one of the reasons participation in this program was voluntary.

Lack of faculty involvement, however, does not appear to have detracted from the perceived value of this teaching program, as NPLs found the program to still be valuable. Although mock-OSCEs, session handouts and teaching tools were not required to be referenced in literature or verified by academic experts, learners reported finding these tools useful for reinforcement of knowledge. This teaching program was designed as an adjunct to rather than replacement of faculty-led teaching, which was reflected in student responses which agreed near-peer teaching in this setting, is a useful supplement to existing education.

While learners did identify areas where both parties were less knowledgeable, they felt these areas were not extensively covered in faculty based teaching either. Furthermore, learners also observed their NPTs appropriately admitted areas where they had limited knowledge. In these situations, NPTs would often allude to their inexperience and recommend NPLs seek clarification with senior academic staff. In order to maintain quality of education and consider integration into formal curriculum, it could be argued that resources created by NPTs should be peer-reviewed and standardised. Furthermore, NPTs could also be given a mandatory list of topics to cover over the course of the year, to prevent areas of weakness in the curriculum from being neglected. These changes however may limit the benefits of cognitive and social congruence and NPTs’ ability to tailor teaching to their desired level of difficulty. Forcing areas of relative weakness may also result in incomplete or incorrect content being taught and result in NPT insecurity around teaching those topics.

Several studies have also suggested and involved monitoring of sessions by academic staff to provide support and clarify doubts, as an alternative.^10^^,^^22^ The disadvantage of routine supervision by expert academic staff is the loss of the low-stress learning environment created by the social congruence between NPTs and learners. In addition, in most studies, teaching quality was also optimised by training NPTs in the necessary skills prior to undertaking their teaching roles. Blank and colleagues provided initial faculty-led training to NPTs to provide accreditation of their skills in physical examination followed by ongoing NPT supervision.^10^ While topics were pre-determined, teaching methods and tools in the different teaching groups were at the discretion of the senior physicians and NPTs.^10^

Despite this, results of this study reveal NPTs felt adequately prepared for their role as NPT, despite minimal senior supervision. Final year students, having recently experienced summative OSCEs, have more recent knowledge of exam expectations and thereby cognitive congruence to assist in preparing junior students for similar exams. Observed confidence in teaching in this program could further be attributed to the unique style of this program giving NPTs freedom to develop session content and thereby teach to their strengths. Furthermore, all NPTs reported prior teaching experience, which could be a result of selection bias due to the voluntary nature of participation in this program. This confidence with teaching may not be reflected amongst all final-year medical students, and subsequently, participation in the program should continue to be voluntary.

In addition, NPTs already receive clinical skills teaching and bedside tutorials with senior staff as final year medical students. Initial NPT training and provision of peer-reviewed teaching resources to guide teaching preparation could improve quality of content taught in our program. A suitable balance of faculty guidance and NPT-led teaching would maximize the benefits and minimize the weaknesses of both strategies.^3^^,^^7^^,^^10^^,^^12^

### Future directions

Current data on peer-education is mainly qualitative. While quantitative evaluation is limited in literature, existing studies suggest peer-education is at least equal to traditional teaching^5^^,^^10^^-^^12^. Given peer-teachers are trainees themselves however, concern has been raised regarding the quality of content taught by NPTs compared to experienced academics.  Researchers Tolsgaard et al.,^12^ Burke et al.^11^ and Blank et al.^10^ conducted separate peer-led teaching programs to teach clinical skills with control groups for comparison.^10^^-^^12^ End-of-program assessment revealed at least equal results when intervention groups were compared with control groups. Peer-educated students additionally felt more confident and had more positive reports regarding their teachers when compared with the control groups.^10^^-^^12^ Moreover, Blank et al.^10^ found students who participated in additional NPT-based teaching convincingly scored better than their peers (p<0.001) in their summative OSCEs.^10^ Further quantitative comparison between peer-led and traditional teaching is warranted to support this and assess if other domains of medical teaching other than clinical skills show similar results.

Ten Cate and Durning^5^ suggest several reasons for implementing peer-education. Not explored in this NPTP is potential benefits to faculty in alleviating teaching burden. Tertiary medical faculties could potentially better allocate their time and resources with the support of adjunctive peer-teaching programs.^5^ Multi-source feedback is an increasingly popular form of training and formative assessment in tertiary education.^5^ Given postgraduate medical training is often structured around an outcome-based curriculum, the provision of constructive feedback to peers is an important skill.^5^ This is something that could potentially be integrated into undergraduate medical training.

Overall students’ perception of the value of this pilot program was positive both before and after summative assessment. Unfortunately, this study was unable to assess if this perceived benefit translated to improved OSCE scores. Comparison of average OSCE results between this cohort and past years, as well as performance of students in MUPCS versus other clinical sites would have provided valuable objective measure of the effectiveness of our POTP.

While this was not possible in this pilot study due to faculty restrictions in sharing sensitive information, future studies could ideally include analysis of summative OSCE results. An alternative would be inclusion of results from faculty run mock-OSCE assessments, conducted at the end of the academic year. This may allow objective comparison of MUPCS students’ performance with students of other Monash University clinical schools who do not offer similar programs but otherwise share a curriculum.

## Conclusions

In summary, peer-led education is valued by peer-teachers and peer-learners alike. The NPLs in our program expressed increased confidence when approaching their final summative OSCE exams. Peer-educators on the other hand enjoyed the opportunity to develop their teaching skills, citing mutual benefit and gratitude to past peer-educators as the strongest motivators for being involved in teaching. Based on the findings of our study and available literature, the authors support the idea that peer-led education would be a valuable adjunct to current medical school curriculums; however participation in these should remain voluntary. The unique features of this program including its goal to improve students’ perceived summative exam performance, duration over one year and completely student-driven initiative with minimal faculty supervision, may be of interest to medical educators wishing to implement similar programs. Furthermore, this paper provides insights into peer-educators’ perspectives which is an identified gap in the literature. Finally, the authors believe that peer-education programs have the potential to develop a culture of teaching in doctors.

### Acknowledgements

The authors would like to thank A/Prof Patrick Fiddes, A/Prof Peter Barton and Dr Phillipa Corby of MUPCS, who supported the running of this project. Your encouragement, enthusiasm and guidance provided us with the opportunity to challenge ourselves by creating this program.

### Conflict of Interest

The authors declare that they have no conflict of interest.

## References

[1] Rashid MS, Sobowale O, Gore D (2011). A near-peer teaching program designed, developed and delivered exclusively by recent medical graduates for final year medical students sitting the final objective structured clinical examination (OSCE).. BMC Med Educ.

[2] Andrew Jay E, Starkman SJ, Pawlina W, Lachman N (2013). Developing medical students as teachers: an anatomy-based student-as-teacher program with emphasis on core teaching competencies.. Anat Sci Educ.

[3] Whitman, Neal A. Peer teaching: to teach is to learn twice. ASHE-ERIC Higher Education Report No. 4. Washington, DC: Association for the Study of Higher Education; 1988.

[4] Lockspeiser TM, O'Sullivan P, Teherani A, Muller J (2008). Understanding the experience of being taught by peers: the value of social and cognitive congruence.. Adv Health Sci Educ Theory Pract.

[5] Ten Cate O, Durning S (2007). Peer teaching in medical education: twelve reasons to move from theory to practice.. Med Teach.

[6] Du X, Kebreya M, Bishop P (2014). A faculty-facilitated near-peer teaching programme: an effective way of teaching undergraduate medical students.. Med Teach.

[7] Naeger DM, Conrad M, Nguyen J, Kohi MP, Webb EM (2013). Students teaching students: evaluation of a "near-peer" teaching experience.. Acad Radiol.

[8] Nelson CA, Frosch Z, Lapin J, Kogan JR (2014). Facilitating the clerkship transition through near-peer-led 'student reports'.. Med Educ.

[9] Protty MB, Mann J, Mohammed MA, Holder R, Wiskin C (2013). Students as teachers: the impact of a near-peer-led didactic teaching model on tutee confidence.. Med Teach.

[10] Blank WA, Blankenfeld H, Vogelmann R, Linde K, Schneider A (2013). Can near-peer medical students effectively teach a new curriculum in physical examination?. BMC Med Educ.

[11] Burke J, Fayaz S, Graham K, Matthew R, Field M (2007). Peer-assisted learning in the acquisition of clinical skills: a supplementary approach to musculoskeletal system training.. Med Teach.

[12] Tolsgaard MG, Gustafsson A, Rasmussen MB, Høiby P, Müller CG, Ringsted C (2007). Student teachers can be as good as associate professors in teaching clinical skills.. Med Teach.

[13] Evans DJ, Cuffe T (2009). Near-peer teaching in anatomy: an approach for deeper learning.. Anat Sci Educ.

[14] Raupach T, Hanneforth N, Anders S, Pukrop T, Th J ten Cate O, Harendza S (2010). Impact of teaching and assessment format on electrocardiogram interpretation skills.. Med Educ.

[15] Rengier F, Rauch PJ, Partovi S, Kirsch J, Nawrotzki R (2010). A three-day anatomy revision course taught by senior peers effectively prepares junior students for their national anatomy exam.. Ann Anat.

[16] Tang TS, Hernandez EJ, Adams BS (2004). "Learning by teaching": a peer-teaching model for diversity training in medical school.. Teach Learn Med.

[17] Kibble JD (2009). A peer-led supplemental tutorial project for medical physiology: implementation in a large class.. Adv Physiol Educ.

[18] Rodrigues J, Sengupta A, Mitchell A, Kane C, Kane C, Maxwell S, Cameron H, Ross M, Ford M (2009). The Southeast Scotland Foundation Doctor Teaching Programme--is "near-peer" teaching feasible, efficacious and sustainable on a regional scale?. Med Teach.

[19] Wong JG, Waldrep TD, Smith TG (2007). Formal peer-teaching in medical school improves academic performance: the MUSC supplemental instructor program.. Teach Learn Med.

[20] Meller SM, Chen M, Chen R, Haeseler F (2013). Near-peer teaching in a required third-year clerkship.. Yale J Biol Med.

[21] Hall S, Stephens J, Andrade T, Davids J, Powell M, Border S (2014). Perceptions of junior doctors and undergraduate medical students as anatomy teachers: Investigating distance along the near-peer teaching spectrum.. Anat Sci Educ.

[22] Reyes CG, Elizondo-Omaña RE, de la Garza O, Guzmán S (2014). Near-peer teaching (NPT): the importance of process evaluation.. Med Educ Online.

